# Evidence of waste electrical and electronic equipment (WEEE) relevant substances in polymeric food-contact articles sold on the European market

**DOI:** 10.1080/19440049.2015.1009499

**Published:** 2015-02-17

**Authors:** Franky Puype, Jiří Samsonek, Jan Knoop, Marion Egelkraut-Holtus, Markus Ortlieb

**Affiliations:** ^a^ Institute for Testing and Certification, Inc., Zlin, Czech Republic; ^b^ Shimadzu Europa GmbH, Duisburg, Germany

**Keywords:** plasma spectroscopy, inductively coupled plasma optical emission spectroscopy, thermal desorption, pyrolysis mass spectrometry, brominated flame retardants, polymer recycling, food-contact materials, waste electric and electronic equipment, waste management, attenuated total reflectance Fourier transformed infrared spectroscopy, X-ray fluorescence, rare earth elements

## Abstract

In order to confirm the possibility that recycled fractions from the waste electrical and electronic equipment (WEEE) stream were illegally entering the European market in black polymeric food-contact articles (FCAs), bromine quantification, brominated flame retardant (BFR) identification combined with WEEE-relevant elemental analysis and polymer impurity analysis were performed. From the 10 selected FCAs, seven samples contained a bromine level ranging from 57 to 5975 mg kg^−^^1^, which is lower than expected to achieve flame retardancy. The BFRs that were present were tetrabromobisphenol A (TBBPA), decabromodiphenylether (decaBDE), decabromodiphenylethane (DBDPE) and 1,2-*bis*(2,4,6-tribromophenoxy)ethane (BTBPE). Typical elements used in electronic equipment and present in WEEE were detected either at trace level or at elevated concentrations. In all cases when bromine was detected at higher concentrations, concurrently antimony was also detected, which confirms the synergetic use of antimony in combination with BFRs. This study describes also the measurement of rare earth elements where combinations of cerium, dysprosium, lanthanum, neodymium, praseodymium and yttrium were detected in four of the seven BFR-positive samples. Additionally, polymer purity was investigated where in all cases foreign polymer fractions were detected. Despite the fact that this study was carried out on a very small amount of samples, there is a significant likelihood that WEEE has been used for the production of FCAs.

## Graphical Abstract

**Figure:**
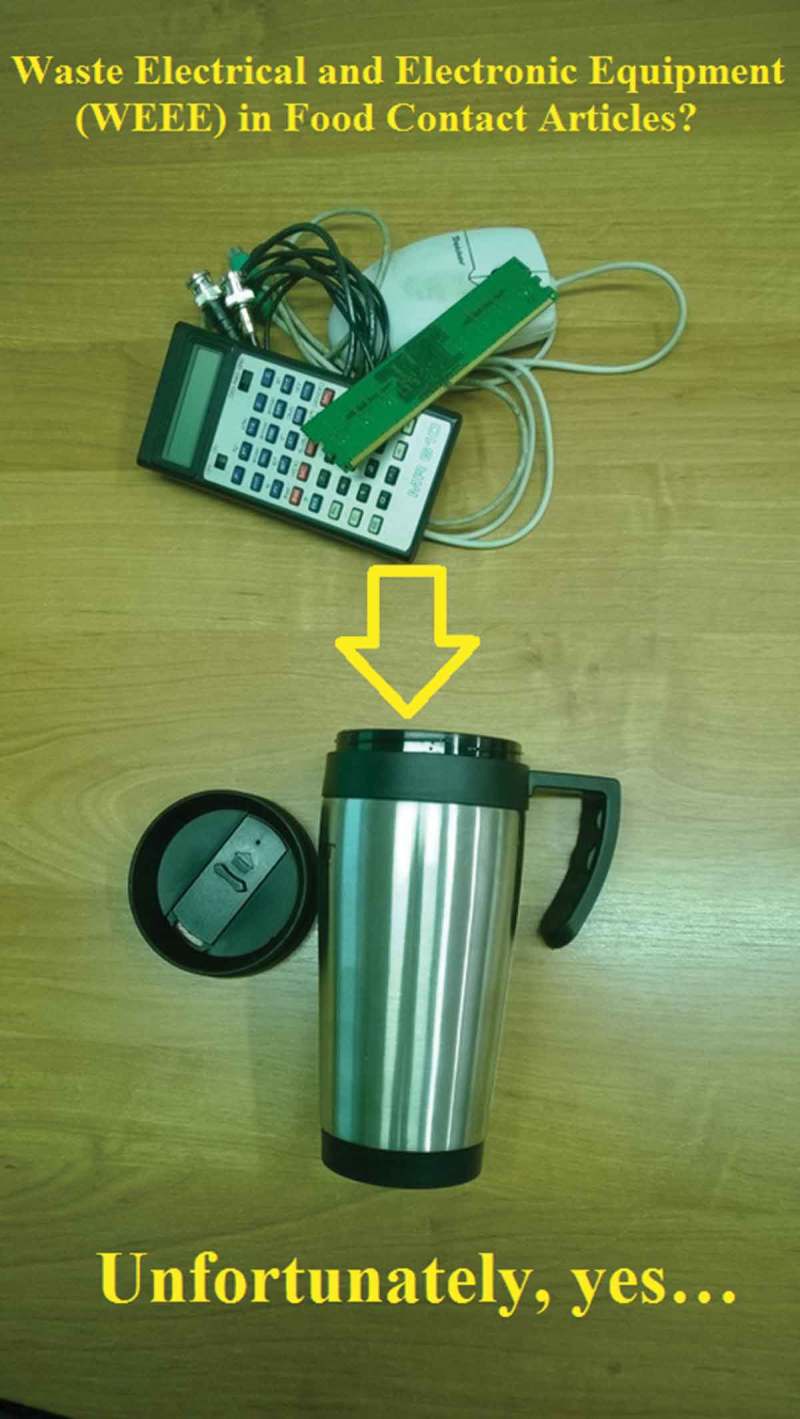

## Introduction

Polymeric food-contact articles (FCAs) are an important group of products that directly influence the quality of food and drinks by releasing a certain amount of chemical constituents by migration. All polymeric materials and polymeric articles intended to come into contact with food such as packaging materials, cutlery and dishes, kitchen processing machines, food containers and materials/articles in contact with water for human consumption fall under the definition of polymeric FCAs. The polymeric material used for the production of such an articles should be preferable food grade and the usage of technical-grade polymers or recycled polymeric waste streams should be avoided as such. As an exemption, reusage of polyethylene terephthalate (PET) is assumed to be safe as the PET cycle is a contained in a closed-loop recycling process (European Commission 2008; Hopewell et al. 2009). Polymeric FCAs are regulated within the European Union by European Commission Regulation 10/2011 (European Commission 2011a). This regulation describes an overall migration test using defined food simulants measuring the amount of non-volatile substances released from a material or FCA. This overall migration test might be interpreted as the measure for the inertness of the FCAs releasing not more than 10 mg of constituents per 1 dm^2^ as the maximum permitted limit. These non-volatile substances might be generally monomers, starting substances, oligomers, colorants, additives or surface additives that are basic constituents of the polymeric FCA, which, if migrating into food, result in an unacceptable change to the food. Requirements laid down by this regulation comprise also the so-called ‘positive list’, which is a list of authorised monomers, other starting substances, macromolecules, additives and polymer aids. All chemicals listed here were individually evaluated for their toxicity and migration behaviour by EFSA which defined a specific migration limit for controlled migration and evaluation of the FCA. The positive list is still expanding as new toxicological data on substances are obtained (European Commission 2012, 2014a). So far, brominated flame retardants (BFRs) are not listed in this positive list and, as a consequence, they are not allowed to be used as initial substances for the manufacture of FCAs sold on the European market. Moreover, as another example, BFRs are regulated within the European Union in food from animal origin, as described in Commission recommendation of 3rd March 2014 on the monitoring of traces of BFRs in food (Commission Recommendation 2014b). BFRs like polybrominated diphenyl ethers (PBDEs) and 1,2,5,6,9,10-hexabromocyclododecane (HBCD) should not exceed the trace level of 0.01 ng g^−1^ wet weight in food from animal origin; tetrabromobisphenol A (TBBPA), tetrabromobisphenol A *bis*(2,3-dibromopropyl)ether (TBBPA-DBPE) and bromophenols should not exceed 0.1 ng g^−1^ wet weight in foodstuffs from animal origin. The reason for these limits is based on findings from EFSA and by the European Commission that many BFRs are persistent, bioaccumulative, and toxic to both humans and the environment.

BFRs are generally used in electronic equipment with the aim to inhibit, suppress or delay the production of flames and as a consequence to prevent the spread of a fire. In the European Union, BFRs present in consumer goods are regulated by the Restriction of the Use of Certain Hazardous Substances in Electrical and Electronic Equipment – the RoHS Directive (European Commission 2011b). According to the directive, electronic equipment resulting at their end of life into waste electrical and electronic equipment (WEEE) should not contain polybrominated biphenyls (PBBs), PBDEs, Hg, hexavalent chromium and Pb any higher than 0.1 weight % and Cd not higher than 0.01 weight %. Through this regulation BFRs like PBBs were completely banned, while for PBDEs only technical decaBDE was allowed (with a ban of pentaBDE and octaBDE). Recently an update of the RoHS Directive was issued (European Commission 2011b), the so-called RoHS2 Directive, which also bans technical decaBDE at a level of 0.1 weight %. The RoHS2 Directive proposed HBCD as a substance of high concern and it is expected that HBCD will be regulated in WEEE as a priority, this being due to the risks to human health and the environment arising from use. The second and complementary law in Europe on the presence of BFRs in consumer goods, the Regulation on Registration, Evaluation, Authorisation and Restriction of Chemicals (REACH) (European Commission 2006), contains the regularly updated Candidate List of Substances of Very High Concern (SVHC; under REACH article 59) restricting HBCD in consumer goods, mixtures and substances (< 0.1 weight %) except for expanded polystyrene (PS) used in the building industry. Rather than a ban, brominated substances are limited by these European regulations, but not completely phased out. While RoHS regulates BFRs with the focus on waste and on homogenous material, REACH regulates BFRs in substances, mixtures and articles but not in waste.

As a reaction to legislation like RoHS and REACH, many producers of flame retardants (FRs) started to distribute alternative FRs to the regulated ones. For example, the demand for decabromodiphenyl ethane (DBDPE) increased in the same period as the demand for decaBDE decreased drastically; therefore DBDPE is generally accepted as the alternative substitute for decaBDE (Ricklund et al. 2008; Bergman et al. 2012; Egebäck et al. 2012). Also the use of halogen-free alternative phosphate-based flame retardants (PFRs) is increasing (Schartel 2010), which in some cases are also used as plasticisers (Brandsma et al. 2014).

This current paper describes a prolonged study based on previous published work (Samsonek & Puype 2013) where the authors focused mainly on the detection of BFRs in thermo-cups and several kitchen utensils purchased on the European market with special attention to black polymeric parts. The presence of BFRs in these black-coloured FCAs suggested contamination by WEEE, this being due to the fact that the bromine (Br) concentrations determined in these products was too low to offer effective flame retardancy and mainly that FCAs have no reason to be made FR due to their use. Therefore the authors suspected inadequate waste stream management introducing the WEEE stream into raw material plastics. Due to the fact that BFRs were detected in food-contact materials and the detected BFRs are not listed in the positive list (Annex 1) of European Commission Regulation (EC) No. 10/2011, this practice is illegal within the European Union. For this current study, the authors wanted to investigate in more detail the suggestion that polymeric FCAs might be contaminated with WEEE streams and are nowadays sold on the European market. By focusing on present WEEE relevant elements in black polymeric FCAs and relating their concentration profile with the detected BFRs, additional evidence for the root of contamination can be demonstrated. Initially, the Br content in the samples was measured by X-ray fluorescence (XRF) analysis followed by BFR identification for the Br-positive samples using thermal desorption GC-MS. In addition, for the quantification of WEEE-relevant elements, inductively coupled plasma optical emissions spectroscopy (ICP-OES) was used. Definitely a key element in electronic applications is Sb which is generally added to polymers as Sb_2_O_3_ involving a highly efficient FR system in combination with halogen-containing FRs. Additionally non-rare earth elements (non-REEs) of interest were selected: As, Be, Cd, Cr, Cu, Fe, Hg, Ni, Pb, Sb and Zn. All these elements have a function in electro and electronic equipment. Also measurements of selected rare earth elements (REEs) like Ce, Dy, Er, La, Nd, Pr, and Y were performed. The criteria to choose these REEs come from their abundance in WEEE based on the demand from industry and the available literature. The applied ICP-OES method for the quantification of REEs in complex matrixes in this paper was previously published by Knoop et al. (2014). The priority of their study was to exclude interfering element emission lines from the target REE emission lines, as the amount of interfering elements (e.g., Al, Cr, Cu and Pb) was in the single per cent range. In the end, 10 analytical emission lines remained from the 280 investigated emission lines suitable for the quantification of REEs in WEEE. Additionally other elements can be determined in different concentration ranges, as the ICP-OES technique benefits from a large dynamic range.

To date this is the first paper reporting the presence of BFRs, WEEE-related elements (including REEs) as WEEE precursors present in polymeric FCAs on the European market. Additionally, to confirm the root of contamination, the polymer matrix and potential macromolecular contaminants were identified by combining attenuated total reflectance Fourier-transformed infrared spectroscopy (ATR-FTIR) and pyrolysis GC-MS. This combined method enabled the efficient detection of impurities from foreign polymer fractions.

### Current status

This paper describes three different ways for proving the likelihood that WEEE has been used for the production of FCAs: the presence of BFRs in FCAs followed by the presence of WEEE-related elements and, finally, the identification of macromolecular contaminants. In order to explain the background to these selected parameters, a brief overview of recent published papers is now given. All recent studies were performed using a similar sample preparation method, comparable analytical instrumentation and a focus on equal target analytes as applied in this paper.

### BFRs in WEEE and their abundance in consumer goods

Ballesteros-Gómez et al. (2013) published an FR analysis method using several separation techniques including 30 target analytes (BFRs and PFRs) on two shredded printed circuit board (PCB) samples and two shredded car interior samples from a car recycling park. TBBPA was detected in all samples. Concerning the overall BFR presence, in one of the PCB samples TBBPA and 1,2-*bis*(2,4,6-tribromophenoxy)ethane (BTBPE) were dominantly present in combination with decaBDE-209. Paine et al. (2014) developed a method for the direct detection of BFRs from plastic WEEE by use of liquid extraction surface analysis mass spectrometry. From shredded and granulated electronic waste samples eight small granulates with different colours, except black, were selected. All samples contained TBBPA as this study was focused on TBBPA. Many up-to-date analytical methods are used for the (semi-)quantification of BFRs in polymers and WEEE in order to prove their use and abundance in daily products (Pöhlein et al. 2005, 2008; Schlummer et al. 2005; Hirai & Sakai 2007; Wu et al. 2007; Vilaplana et al. 2008; Entwisle 2010; Chen et al. 2012). In all cases contamination of the environment by BFRs is directly related to the usage of FRs in consumer goods (Hajšlová et al. 2007; Jenssen et al. 2007; Zhang et al. 2009; Gieroń et al. 2010; Cai et al. 2012). Their presence in the environment results in BFR contamination of the human food chain (Covaci et al. 2006; Fernandez et al. 2007; Zhao et al. 2007; Schecter et al. 2010; Clement et al. 2012; Miège et al. 2012) with several toxic effects (Szymańska et al. 1999; Darnerud 2003; Harju et al. 2007) as a consequence.

Gallen et al. (2014) studied the overall Br content and BFR presence in 1714 consumer goods of all kinds (automotive parts, electronics, toys and auto-accessories) where they measured 882 (51%) of the samples as Br positive (i.e. > 1 mg kg^−1^) by use of an XRF method. After applying several separation methods they could prove the presence of PBDEs, HBCD and TBBPA in these samples. The decaBDE-209 congener appeared the most frequent of all the PBDE congeners coming from the intensive usage of technical decaBDE. PBDEs are a typical group of FR additives used in electronic equipment and are still expected to occur in plastic waste streams. Hosaka et al. (2005) reported a semi-quantitative method by using thermal desorption injection of sample extracts from PS (black television back plate) with tetrahydrofurane in combination with GC-MS detection for the quantification of decaBDE in PS. Additionally, direct thermal desorption of polymer samples was manifested, proving to be quick and effective for BFR screening. This method has also proved to be effective for BFR determination in combination with XRF measurements in order to determine Br in consumer goods and evaluate compliance with present European legislation (Samsonek & Puype 2007; Puype & Samsonek 2008; Stapleton et al. 2011).

There are many variations given in the literature as to the lowest Br concentration ensuring flame retardancy in polymers and it is difficult to decide on an exact concentration. However, from the literature it has been reported that the average or lowest Br content in WEEE can be estimated. Arias (2001) reported that PS can be made FR at a BFR concentration ranging between 0.8 and 4.0 weight % (HBCD), while polyolefins are made flame retardant with PBDE concentrations ranging between 5 and 8 weight %. Another study reports that for expanded PS the addition of HBCD between 0.5 and 1.0 weight % is sufficient to achieve flame retardancy (US National Research Council 2000). Dimitrakakis et al. (2009) reported their measured average Br concentrations detected in small WEEE, which was 5300 mg kg^−1^. Other authors reported the Br content in WEEE ranging between 4300 and 41 000 mg kg^−1^ (Vehlow & Mark 1997), between 4200 and 6800 mg kg^−1^ (Association of Plastic Manufacturers in Europe 1997); or between 150 and 25 000 mg kg^−1^ (Fink et al. 2000).

### Abundance of elements in WEEE

Dimitrakakis et al. (2009) measured elements in a large number of small WEEE (sWEEE) samples (*n* = 161) by use of a portable energy dispersive X-ray spectrometer. The results demonstrated that BFRs were present in approximately half of the plastic samples, which confirms the results reported by Gallen et al. (2014). Moreover, in most of the cases antimony trioxide (Sb_2_O_3_) was detected. Sb_2_O_3_ is generally used as a synergist FR in combination with BFRs (Pitts 1972; Simon et al. 1982). The mean concentration of Br exceeded the level of all other measured non-REEs (Pb, Cd, Hg, Ni, Zn, Cu, Cr, Sb, Fe, Sn, V and As), except for Ti (0.8%), which is a commonly used pigment in polymer applications (TiO_2_). The average Sb content was about 0.2% and no RoHS-regulated elements have been found to exceed the limit values. Jakab et al. (2003) studied the effect of Sb_2_O_3_ addition (5.0%) for two brominated flame retarded high-impact polystyrene (HIPS) samples (one doped with 13.0 weight % DBDPE and the other doped with 13.0 weight % decaBDE). They discovered a higher thermal stability for polymers containing Sb_2_O_3_ and BFRs than the polymers containing only the BFRs at same concentration. In their paper these authors described that the two evaluated BFRs thermally degrade by different pathways: DBDPE decomposes to bromotoluenes, while decaBDE-209 creates brominated dibenzofuranes by an intermolecular ring closure pathway. During the thermal degradation process at a temperature of 370°C, Sb_2_O_3_ is releasing water by H-abstraction from the polymer chains combined with the production of SbBr_3_ after partial debromination of the FRs. For practical reasons, the mixed Sb_2_O_3_ synergist can be used to lower the level of the total required BFR dosing needed to give FR material as an advantage. Generally, Sb_2_O_3_ synergist is added to the polymer in the lower percentage range. Another advantage of using inorganic FRs is the combination of extinguishing properties, as BFRs inhibit gas phase combustion by free radical scavenging, the inorganic FRs (e.g., hydroxides and carbonates) function in the gas phase and the condensed phase by releasing non-flammable substances (H_2_O, CO_2_) which dilute the fuel and cool the polymer surface. Inorganic FRs are forming during combustion a crust on the polymer surface to prevent access to oxygen by the polymer fuel. Other commercial inorganic (synergist) FRs are iron oxides, zinc borates, zinc stannate, zinc phosphate, magnesium hydroxide, aluminium hydroxide, calcium carbonate and magnesium carbonates (Pritchard 1998). Wienold et al. (2011) studied the sampling procedure for RoHS-relevant elements (Cd, Hg and Pb) in ground PCBs. They evaluated several digestion procedures on several particle sizes. The overall outcome was that already for a particle size of less than 1.5 mm a sufficient analytical result can be obtained. The influence of the several acid digestion procedures (open vessel digestion and pressurised microwave digestion) using several acid combinations on relevant element concentrations did not affect the analytical result ascertainably. A good sampling procedure in this case is crucial as such matrixes have generally a strong inhomogeneity as a drawback.

Ferrous metals in electric equipment are mainly used for castings or as major elements in magnets and magnetic coils. Be is used mainly in metalloid applications like Be/Cu alloys, beryllium oxide-ceramic or as metallic Be. Cu is a prominent element in cabling and connectors (UNEP REPORT 2013). Besides the suspected presence of non-REEs as WEEE-relevant elements, the industrial use of REEs is also linked to applications in electronics and, therefore, might be present in WEEE-contaminated FCAs. Oppermann et al. (2013) studied the presence of REEs in completely milled and digested WEEE samples (mobile phones, LCD screens and seven circuit boards). They detected by ICP-OES measurement concentrations of Nd, La, Pr, Dy, Y, Er and Ce ranging from 1 to 100 mg kg^−1^. In all samples Y was detected following by Ce as the most abundant present REEs (in three samples). REEs like Ce, Dy, La and Y are used in light-emitting diodes (LEDs); Ce, La and Y are used in fluorescent powder from lighting components and televisions; batteries from consumer goods like cameras and laptop computers contain Ce, La, Nd and Pr; Nd is a specific element used for permanent magnets in microphones, professional loudspeakers, in-ear headphones and computer hard disks; Pr is used in some applications together with Nd due to their excellent magnetic properties (Goodship & Stevels 2012). REEs have strongly limited resources and so mining and subsequent processing is always a very critical step, as the purification of the REEs containing ores requires the use of high amounts of chemicals. For this reason, REEs are classified as a raw material with the highest level of supply risk (European Commission 2010).

### Abundance of polymeric materials in WEEE

Concerning the most abundant polymeric materials in the sWEEE stream, Martinho et al. (2012) published an overview of polymers by direct sampling from a recycling unit in Portugal by using a portable near infrared device. From the 3417 samples (large cooling devices, sWEEE, copying equipment, printers, cathode ray tube devices, etc.) the most abundant types of polymers were PS, acrylonitrile–butadiene–styrene copolymer blends (ABS), bisphenol A (BPA)-based PC or PC/ABS, HIPS and polypropylene (PP). The study also describes the colour of the samples where the cathode ray tube monitors (73%) and sWEEE (22%) were evidently darkly (black or brown) coloured.

## Materials and methods

### Sampling, statement and testing strategy

All 10 black polymeric FCAs were purchased randomly from different distributors within Europe between 2012 and 2013. From this rather small group of samples, three samples were typical kitchen utensils, while seven samples were used as an upper part of a thermo-cup for storage of hot drinks. These upper parts from thermo-cups (closure lids) were chosen as they come into direct contact with the mouth and are intended to be used for hot drinks where a higher rate of migration of contaminants into the food/drink is expected. All samples were stored in darkness to avoid photolysis of the BFRs. Only black items were selected due to the suspected presence of BFRs. It is suggested that black polymeric items have a high chance of being contaminated by recycled polymers like polymers from WEEE streams. Technically speaking, a melt of recycled polymeric material with virgin material does not look attractive; however, after colour unification with black pigments the black polymeric material looks attractive for the customer. Unfortunately, from the customer’s point of view these FCAs might contain fractions of BFRs made from WEEE.

Beside the fact that this study focused on a small number of samples, this is the first study combining WEEE-related elemental analysis (REEs and non-REEs) with BFR characterisation and specific polymer analysis. Initially samples were screened by XRF for total Br content in combination with thermal desorption GC-MS for the identification of the brominated additives. For the elemental analysis all samples were acid digested and measured by ICP-OES. Small parts of the polymer samples were measured by ATR-FTIR and after a pre-extraction process in toluene and acetone they were applied for pyrolysis GC-MS for detailed polymer identification.

### XRF analysis: screening for Br

This spectral method is very effective for the measurement of Br in plastic materials and has the ability to screen quickly many test points on one sample without a time-consuming sample treatment. Br was taken as the first indication for WEEE contamination due to the fact that BFRs are intensively used in electrical and electronic applications. Despite there possibly being other non-BFRs used, BFRs are still prominently present in the WEEE streams. XRF analysis was used initially to distinguish between Br-positive and -negative samples. The XRF analyses were performed on a Shimadzu EDX-800P spectrometer (Kyoto, Japan) equipped with a Si(Li) semiconductor detector cooled by liquid nitrogen. In order to prove the presence of Br in polymers, the instrument parameters were optimised to measure total Br in a hydrocarbon matrix. The acceleration voltage of the X-ray tube with an Rh target was set to 50 kV with a current towards the filament of approximately 120 µA. The X-ray tube radiation was filtered by a Ag filter (primary filter) for background reduction. The warming up time of the source was 15 s, while the measuring time was set to 100 s. For Br, the K_α_ spectral line at 11.92 KeV is the most sensitive and therefore this was chosen as the analytical line for quantification. Unfortunately, this line can be overlapped by the K_β_ lines from As and also by the L_β1_ and L_β2_ lines from Hg. Such possible overlaps are checked by using the data processing functionality of Shimadzu’s PCEDX software (Version 1.02). Interferences are automatically subtracted from the K_α_ peak of Br if necessary. A similar method is described in an international standard published in 2009 by the European Committee for Standardization (CEN) concerning the analytical support of the RoHS directive, namely standard EN 62321 (CEN 2009). This CEN standard was taken as a guide for Br quantification. For calibration of the instrument, an RoHS standard set of Sumika Chemical Analysis Service, Ltd (Osaka, Japan) was used. This set contains six PE standards with a calibration range of approximately 0–1200 mg kg^−1^ Br (0, 125, 270, 624, 627 and 1214 mg kg^−1^). To obtain intensity data, the peak area between 11.66 and 12.16 KeV was fitted to a Gaussian curve and integrated. Furthermore, internal standard background compensation was applied. The recommended sample size needs to be larger than a 15 mm diameter circle. As a control measurement, two certified reference materials (CRMs) were measured at different Br concentration levels. They were the ERM^®^-EC591 (JRC-IRMM, Geel, Belgium), which is a PBDE/PBB-doped PP in pellet form with an overall Br concentration of 2.08 ± 0.07 g kg^−1^, and ERM-EC680K PP pellets (JRC-IRMM) spiked with inorganic pigments having an elemental Br concentration of 96 ± 4 mg kg^−1^. Samples with a total elemental Br concentration higher than 40 mg kg^−1^ were considered as Br-positive with this concentration as the LOD.

### Thermal desorption GC-MS: identification of BFRs

Complementary to XRF analysis, BFRs were identified by thermal desorption GC-MS. Thermal desorption is a sample introduction method using heat to extract/vaporise additives from a polymer matrix or a sample extract. In this cases sample extracts were made from the selected polymer samples. Smaller pieces of polymer were cut in pieces of about 1 mm^3^ followed by a 24-h static migration in toluene (GC/ECD-grade residue analysis, Chromservis s.r.o., Prague, Czech Republic). Migration was performed at RT in small amber glass vials to avoid photo-degradation of the BFRs. As a practical approach, the added volume of toluene (ml) was twice the sample weight (g) in case the sample was not swelling/solving during the extraction. In case the sample was solving in toluene, a higher amount of toluene was added up to an estimated fivefold of the sample volume in order to get at least 50 µl of toluene extract to be taken from the top by use of volumetric glass micropipettes (Brand, Wertheim, Germany). The supernatant or solved polymer mixture was then transferred into an 80 µl deactivated stainless steel sample cup which on the was inside covered with a thin layer (< 1 µm) of fused silica. Polymers are a quite difficult matrix to quantify additives by classical GC-MS due to the fact that a complex precipitation step has to be carried out to avoid injection of a higher fraction of oligomers into the GC injector causing analyte discrimination and carryover effects. This disadvantage can be avoided by using a thermal desorption system based on sample cups which are removed from the liner space after each analysis by a pressure shot into an external receiver. Toluene as an extraction/dilution solvent has been favoured because most of the commercial BFRs are well soluble in toluene. The sample cup was placed on a 48-position autosampler (auto-shot sampler AS 1020-E, Frontier Laboratories Ltd, Fukushima, Japan) and injected by freefall into the furnace existing of a quartz pyrolysis tube which is placed above the injector of the GC coupled with an interface needle through the septum. The thermal desorption settings were optimised starting from an initial temperature of 150°C with a ramp rate 80°C min^–1^ up to 350°C and a hold time of 2 min. In order to evaluate the cleanness of the whole sample path and to screen potential carryover effects from a previous measured sample, the thermal desorption sequence was programmed so that firstly a blank sample cup without any sample or toluene was injected and measured. Then a sample cup spiked with toluene was injected after evaporation to clarify the cleanness of the used toluene. This measurement is taken as the reference blank measurement and compared with a duplicate measurement of the sample extract. Sequentially a cycle of four measurements is repeated as many times as there are samples. As a critical parameter, the interface temperature from the space between the thermal desorber unit and the GC has been found to be optimal at 300°C with a high and safe yield for decaBDE-209 and the lowest formation of thermal debromination products (heptaBDEs, octaBDEs and nonaBDEs). At higher temperatures thermal degradation of decaBDE-209 occurs with loss of sensitivity as a consequence. For this study, a GC-MS QP2010 Plus from Shimadzu was used equipped with a metal capillary separation column (Ultra ALLOY-PBDE; 0.25 mm inner diameter × 15 m, Frontier Laboratories) coated with a very thin (0.05 µm) film of immobilised-polydimethylsiloxane. The initial column oven temperature was set at 40°C during the thermal desorption process followed by a temperature gradient of 15°C min^–1^ to 315°C and kept for 5 min. The injector split ratio was intentionally programmed high (60:1) in order to have a high carrier gas flow along the sample cup resulting in an efficient transport of the target analytes towards the analytical column. The interface between the MS and the column end was kept at 300°C, while the ion source was kept at 270°C. The applied ionisation mode was electron impact with an ionisation energy of 70 eV. The MS was tuned on perfluorotributylamine so that the peaks had a full width at half maximum value of 0.4 *m*/*z* for low and higher masses. For brominated target analytes such a resolution is favoured due to a better match with the reference library for the identification of higher brominated FRs as an advantage (US National Institute for Standards and Technology, NIST electron impact mass spectral library and Wiley Registry). All peaks were monitored by the fast automated full scan–single ion monitoring technique (FASST), which enables both full-scan (SCAN) and single-ion monitoring (SIM) data to be acquired on one peak. The application of the FASST mode for BFR screening in polymers enables one to make effective conclusions concerning the presence of polymeric BFRs (e.g., oligomers from dibromostyrene copolymer), reactive BFRs (e.g., TBBPA and derivates) or reaction products from thermal debrominated additive BFRs (e.g., tribromobisphenol A from TBBPA).

As the most sensitive part of the FASST mode, the SIM method has been programmed so that most of the actual abundant BFRs could be monitored. For the detection of abundant PBDEs, the SIM method was designed for the use of certified standard solutions containing triBDE-28, tetraBDE-47, pentaBDE-99, pentaBDE-100, hexaBDE-153, hexaBDE-154, heptaBDE-183 and decaBDE-209 purchased from AccuStandard Inc. (New Haven, CT, USA). The whole method was fine-tuned for PBDE screening by use of technical pentaBDE, technical octaBDE purchased from LGC Promochem GmbH (Wesel, Germany) and technical decaBDE mixture (SAYTEX 102E flame retardant) purchased from Albemarle Europe sprl (Louvain-la-Neuve, Belgium). For PBBs a mixture of selected brominated congeners was used to design the SIM method. Therefore a PBB standard solution of monoBB-3, diBB-15, triBB-18, triBB-18, tetraBB-52, pentaBB-101, hexaBB-153, heptaBB-180, octaBB-194, nonaBB-206 and decaBB-209 was purchased from Wellington Laboratories Inc. (Guelph, ON, Canada). For PBDEs as well as for the PBBs the molecular ion [M]^+^ cluster and the electron impact fragmentation ion cluster after debromination [M – Br_2_]^+^ cluster were chosen for identification and confirmation. The simultaneous SCAN method, as the second part of the FASST mode, was designed so that the lowest mass range starting from *m*/*z* 230 is low enough still to detected fragments of HBCD, di-bromotoluene, bromostyrenes, monobromobiphenyl, monobromodiphenylether and dibromophenol, and high enough to avoid oligomeric interferences from polyolefins, styrene-based copolymers (dimers), polyamides (PA) and PC based on BPA. The highest border of the SCAN method at *m*/*z* 1000 was sufficient for the detection of most commercially available BFRs. The scan method was verified with analytical standards: TBBPA from AccuStandard, TBBPA-DBPE from Dr. Ehrenstorfer GmbH (Ausburg, Germany), HBCD from Sigma-Aldrich (Steinheim, Germany), DBDPE-209 from Wellington Laboratories, technical DBDPE (CHITEX FR-940) from CHITEC (Taipei City, Taiwan), BTBPE from Chemtura Corporation (Middlebury, CT, USA), and 2,4,6-tribromophenol (PH-73FF) from Great Lakes Solutions (West Lafayette, IN, USA).

To check the method performance, CRM was measured as a quality standard containing critical BFRs (ERM^®^-EC591). The basic polymer from the ERM^®^-EC591 was PP doped with commercially used BFRs like technical pentaBDE (700 mg kg^−1^), technical octaBDE (200 mg kg^−1^), technical decaBDE (700 mg kg^−1^) and technical decaBB (700 mg kg^−1^). By this applied method no carryover effects were observed; however, from the analytical point of view, this method cannot be used for quantification of BFRs due to the diversity in hardness of the polymer samples, solubility differences in toluene and chemical composition of the different polymer matrixes with various affinity between the BFR and the polymer chain. This method was primary designed as a screening method for common BFRs (target analytes) on the market to identify their degradation products present in the polymers (non-target analytes) and to detect newer BFRs.

### Acid digestion

In order to determine the elements from the polymeric matrix, acid digestion was performed with a microwave-assisted digestion system (Speedwave four with DAK-100/4 PTFE closed vessels; Berghof, Eningen, Germany). For all samples the same sample preparation step was applied because of their comparable composition. To overcome accuracy uncertainties related to homogeneity of the elements in the material, all acid digestions were done in triplicate. A sample weight of 0.25 g was acid digested by adding 8 ml of HNO_3_ (67 weight %; Analpure, Analytica, Czech Republic) and 2 ml of hydrogen peroxide (30 weight %; Lachner, Neratovice, Czech Republic). The sample was cut into small pieces, placed into a 100 ml vessel which was pressurised to 75 bar and heated towards 230°C within 20 min. This temperature was kept for another 55 min. Each sample was joined with a sample blank with a similar acid composition for comparison/subtraction of background signals. In order to evaluate the acid digestion step, two CRMs were acid digested. They were ERM-EC591 (JRC-IRMM), which is PP in pellet form containing Sb_2_O_3_, and ERM-EC680K (JRC-IRMM), which is also PP in pellet form containing As, Cd, Cr, Hg, Pb, Sn, Zn and Sb. For all elements the recovery fell within the 80–120% interval for a triple measurement. Note that there was no CRM available for polymeric WEEE containing all target REEs. Therefore for the evaluation of the measurement on REEs another measurement procedure was applied (see below).

### ICP-OES analysis: element concentrations

The selected elements for monitoring were As, Be, Cd, Cd, Cu, Cr, Fe, Hg, Ni, Pb, Sb and Zn. For the analysis of REEs Ce, Dy, Er, La, Nd, Pr and Y were selected due to their abundance in WEEE. All measurements were performed by ICP-OES (ICPE-9820; Shimadzu). The advantage of using simultaneous ICP-OES is that the analytical plasma can be observed axially and radially. Both plasma observations can be combined in a single method, which means traces can be measured using a real axial view and major elements for the same sample by using a real radial view. Special attention should be taken for the possible spectral interferences, especially for the elements present at trace levels. Also the RoHS-regulated elements were measured for the purpose of this study.

For the measurement of REEs, at first, spectral interferences between the analytes themselves were considered. This was evaluated by using an external-permuting calibration model, which does not calibrate with an equal rising concentration for all elements, but mixes the elements at several levels randomly. By this calibration strategy the use of interfering spectral lines can be eliminated. Moreover the evaluation of REEs analytical spectral lines/analytical lines has been done in solutions containing high levels of Fe, Al, Ti, Cr, Mn, Co, Ni, Cu, Zn, Zr, Nb, Ag, Sn, Au and Pb, respectively. Each element has been measured by use of at least two spectral emission lines. For the other elements more spectral emission lines were selected in order to confirm the absence of spectral interference. Sample blanks were treated like the samples and were taken into account for the final concentration calculation. After all interferences were excluded. An REEs solution (VHG Labs, Manchester, NH, USA) was used to perform daily calibrations. Single-element solutions and mixed single-element solutions (Merck KGaA, Darmstadt, Germany) were used for all other calibrations. For major elements (Cr, Cu, Sb) with suspected interferences, decadal dilutions (1:1; 1:10; 1:100; 1:1000) were used by checking the signal. By measuring each dilution, a linear fit should be observed within the calibration range. The hydride-forming elements at trace levels (Hg, As) were analysed by using the hydride vapour generator technique (HVG) by using the HVG-1 device (Shimadzu) connected to the ICP-OES. By this technique in a first step the sample pH is assured, which is an acidified environment by use of HCl (206 ml of 30% HCl, Merck KGaA, per 500 ml of deionised water). The second step is the formation of elementary Hg and hydrides of As by coming into contact with a solution of sodium borohydride (2.0 g NaOH and 2.5 g of NaBH_4_, Merck KGaA, per 500 ml of deionised water). The gas phase is separated from the liquid phase and transported to the plasma. The great benefit of this technique is the separation of analyte vapours from the other contents of the sample. For example, As can be analysed using the sensitive 228.812 nm line. Typical spectral interferences caused by Cd (228.802 nm) can be excluded, as Cd does not form hydrides and is separated into the waste. Additionally, this method is more sensitive as by standard sample aspiration 2–4% of the sample is considered (the sample aerosol is transported to the plasma) and by the HVG technique all the formed Hg and As hydrides were measured without any losses. To determine method sensitivity regarding each element, the 3*σ* criteria of blank measurement was used for the estimation of LOD. Table 1 shows all the analytical lines with further information such as LOD or observation and the measurement technique.

### ATR-FTIR analysis: polymer identification

ATR-FTIR analysis was performed for the identification of the polymer matrix. All identification was carried out on a Shimadzu IRPrestige FTIR spectrophotometer equipped with a single reflectance diamond ATR crystal. The measuring range was 4600 to 400 cm^−1^ at 20 scans per sample with a wave number resolution of 2 cm^−1^ by using the Happ–Genzel transformation as the apodisation function. The water peaks were subtracted using atmosphere correction mode. All spectra were compared with standard spectra from databases that are commercially available, e.g. RoHS, ATR-Polymer2, IRs Polymer2 and T-Polymer2, all running on LabSolutions IR software (Shimadzu) combined with in-house libraries. For the identification of the main polymers, the match with the libraries was 90% or higher.

### Pyrolysis-GC-MS for the identification of macromolecular impurities

In addition to identifying contamination from foreign polymer waste streams, pyrolysis analysis was performed. Pyrolysis coupled with GC-MS is an instrumental method that enables reproducible characterisation of (co-)polymers either as a majority or as a trace contamination. The method detects monomers and the bigger pyrolysis degradation products (oligomers and their specific chromatographic pattern) to obtain a full compositional overview of the polymer matrix. The model of pyrolytic reactions in plastics is dependent on the kind of polymer, and with this background each single reaction step is representative of a complex network of reactions. Radical chain pyrolysis is a common reaction for polyolefin-type polymers where radicals are formed and induce a homolytic scission with the carbon in the β-position evolving the initial monomer (e.g., styrene in PS). Many authors describe this depolymerisation process also as the unzipping reaction due to its sequential repetition. Beside the β-scission, polyolefins also undertake subsequent intra- and intermolecular hydrogen transfer resulting in alkanes and dienenes. The combination of pyrolysis reactions results in a remaining mixture of saturated and unsaturated fragments which give a very specific pyrogram (Bockhorn et al. 1999; Ballice & Reimert 2002). By a similar mechanism, PS blends and co-polymers like styrene–acrylonitrile co-polymer (SAN) and ABS fragment by a similar mechanism, however, the styrenic part favours mainly a β-scission reaction resulting in a high quantity of unzipped styrene, α-methylstyrene, styrene dimers and trimers (Rutkowski & Levin 1986; Westerhout et al. 1997; Achilias et al. 2007). Acrylic polymers have similar fragmentation patterns as the acrylate esters appearing in the pyrograms are the most abundant (Straus & Madorsky 1953; Wallisch 1974). Condensed polymers like PA, PET, polybutylene terephthalate (PBT) and PC can also be evaluated by pyrolysis GC-MS. However they release their initial monomers to a lesser extent. This due to the fact that these polymers have reactive places around the oxygen atom where reactions like transesterification, radical scission, cyclisation, H-abstraction and water abstraction commonly appear. In the case of PET or PBT, benzoic acid and 1-phenylpropane from the terephthalate degradation were the main target analytes (Oguri et al. 1992). For PC comprised of BPA, several alkylphenolic derivatives might appear (Oba et al. 2000; Becker et al. 2001). PA6 releases mainly caprolactam after intramolecular exchange (Czernik et al. 1998). After pyrolysis the evolved analytes are swept onto the analytical column (initial temperature 40°C) and GC-MS analysis proceeds as normal (15°C min^–1^ to 320°C for 10 min). For this analysis, the pyrolyser was programmed at 650°C with He as the carrier gas. Up to 10 mg of the toluene/acetone, pre-extracted sample was injected. An intensive pre-extraction step was required in order to keep the matrix additive-free as additives might interfere in the resulting pyrogram. The hardware configuration was the same as used for the thermal desorption GC-MS experiment (PY-2020iD, Frontier Laboratories/GC-MS QP2010 Plus, Shimadzu), however with a required improved separation of the volatile degradation products using the Ultra ALLOY-5 column (0.25 mm i.d.× 30 m × 0.25 μm film thickness; Frontier Laboratories). The mass spectrometer was programmed in full-scan mode from *m*/*z* 50 to 1000 with electron ionisation (70 eV). Peaks from the pyrograms were identified by using the NIST 05 library with a minimal match of 80% in combination with retention time comparison and oligomeric distribution patterns from pyrograms of known polymer matrixes. Similar to the thermal desorption GC-MS analysis, all measurements were repeated twice and compared with blank measurements to check the cleanness of the system and to avoid potential carryover problems. Depending on the expected macromolecular contaminations, several target analytes were selected for screening (Table 2). In this paper the following target polymers were measured: HIPS, SAN, ABS, PBT, PET, PA6, PS, polymethylmethacrylate (PMMA) and PP/polyethylene (PE). In many cases there were more possibilities to define the polymer structures. As an example, if in the pyrogram styrene was detected, there are a few possibilities left (e.g., PS/HIPS/ABS/SAN) to postulate which might be reduced by detecting 1,4-butadiene, 4-ethenyl-cyclohexene (cyclic dimer from butadiene), 4-cyano-cyclohexene (cyclic dimer of butadiene-acrylonitrile) and α-methylstyrene (from polymerised PS) into the presence of ABS as a final conclusion. Therefore, in some cases the exact identification of macromolecules might be under discussion. Before discussing such an issue, in our opinion and findings the goal is to detect foreign polymeric material in the main polymer matrix, which leads to the suggestion of WEEE contamination.

**Table 1.** LOD values for the selected elements with wavelengths and basic measuring parameters.

Element	Quantification wavelength (nm)	Plasma	Transport to the plasma	LOD (mg kg^−1^)	Confirmation wavelength(s) (nm)
As	189.042	Axial	Hydride vapour technique	0.10	193.759
Be	313.107	Axial	Coaxial nebuliser	0.0074	–
Cd	214.438	Axial	Coaxial nebuliser	0.069	–
Ce	446.021	Axial	Coaxial nebuliser	0.27	–
Cr	205.552	Axial	Coaxial nebuliser	0.074	267.716
Cu	213.598	Axial	Coaxial nebuliser	0.26	224.700
Dy	353.170	Axial	Coaxial nebuliser	0.061	–
Er	349.910	Axial	Coaxial nebuliser	0.019	369.265
Fe	234.349	Axial	Coaxial nebuliser	0.11	238.204 and 259.940
Hg	184.950	Axial	Hydride vapour technique	0.0034	194.227 and 253.652
La	399.575	Axial	Coaxial nebuliser	0.027	–
Nd	406.109	Axial	Coaxial nebuliser	0.030	417.732
Ni	231.604	Axial	Coaxial nebuliser	0.10	–
Pb	220.353	Axial	Coaxial nebuliser	0.76	–
Pr	417.939	Axial	Coaxial nebuliser	0.62	–
Sb	206.833	Axial	Coaxial nebuliser	1.75	–
Y	371.030	Axial	Coaxial nebuliser	0.016	377.433
Zn	202.548	Axial	Coaxial nebuliser	0.06	206.200 and 213.856

**Table 2.** Overview of selected target analytes appearing in the pyrograms for characterisation of macromolecular contaminants.

Target analyte	Retention time (min)	Target mass (*m*/*z*)	Polymer
1,4-Butadiene	1.1	53.0/54.1	HIPS/ABS
Methacrylic acid methylester	2.5	69.0/100.0	PMMA
4-Ethenyl-cyclohexene	3.91	54.1/79.1/93.1/108.0	HIPS/ABS
Styrene	4.9	78.0/104.1	PS/HIPS/ABS/SAN
α-Methylstyrene	6.1	103.1/118.2	PS/HIPS/ABS/SAN
4-Cyano-cyclohexene	6.3	54.1/67.1/79.1/92.1/107.1	ABS/SAN
Benzoic acid	8.0	77.0/105.1/122.1	PBT/PET
Caprolactam	9.0	55.0/85.1/113.1	PA6
1-Phenyl-1-propane	10.1	54.1/77.0/105.1	PBT/PET
Homologous series of aliphatic alkanes/alkenes/dienes	Sequential triplets or repeating distributions	55.1/57.1/69.1/ 71.1/83.1/85.1	PP/PE

**Table 3.** Sample list with sample description and results showing the macromolecular contaminants, Br content and BFR identification.

Sample number	Sample	Colour	Main polymer^a^	Detected monomers (pyrolysis GC-MS)	Macromolecular contamination^a^	Br content (mg kg^−1^)^b^	Detected BFRs^c^
							
1	Egg cutter	Black	PP/PE	4-Ethenyl-cyclohexene; styrene; α-methylstyrene; benzoic acid	HIPS/PBT or PET	57	TBBPA, decaBDE
2	Electric frying pan	Black	PBT	1,4-Butadiene; 4-ethenyl-cyclohexene; styrene; α-methylstyrene	HIPS or ABS or SAN	5975	TBBPA, DBDPE
3	Apple cutter	Black	ABS	Methylmethacrylate	PMMA	279	TBBPA, DBDPE, BTBPE
4	Screwable part (thermo-cup cover)	Black	PP/PE	Styrene; α-methylstyrene	PS/PBT or PET	66	TBBPA, decaBDE
5	Movable lid (thermo-cup cover)	Black	ABS	Methylmethacrylate; benzoic acid	PMMA/PBT or PET	504	TBBPA, decaBDE
6	Screwable part (thermo-cup cover)	Black	PP/PE	Benzoic acid; 4-ethenyl-cyclohexene styrene; α-methylstyrene	PBT or PET HIPS or ABS or SAN	n.d.	n.d.
7	Movable lid (thermo-cup cover)	Black	ABS	Methylmethacrylate	PMMA PC PP	1521	TBBPA, decaBDE, DBDPE
8	Screwable part (thermo-cup cover)	Black	PP/PE	Benzoic acid; styrene; α-methylstyrene	PBT or PET PS	n.d.	n.d.
9	Screwable closure (thermo-cup cover)	Black	PP/PE	Benzoic acid; 4-ethenyl-cyclohexene; styrene; α-methylstyrene	PBT or PET HIPS or ABS or SAN	62	TBBPA, decaBDE
10	Screwable closure (thermo-cup cover)	Black	PP/PE	Methylmethacrylate; styrene; α-methylstyrene	PMMA PS PBT or PET	n.d.	n.d.

Notes: ^a^The main polymer and possible polymeric contaminants were interpreted from FTIR spectra combined with pyrolysis GC-MS data.

^b^Measured by XRF; n.d., not detected and implements an LOD of 40 m g kg^−1^.

^c^Measured by thermal desorption GC-MS.

## Results and discussion

Initially the presence of Br and BFRs was investigated in the black selected sample parts. From the 10 selected samples, seven samples contained BFRs, however at a lower Br level than suggested to achieve sufficient flame retardancy (for results, see Table 3). The highest Br level was found in a PBT sample with 5975 mg kg^−1^ Br from TBBPA and DBDPE, while the lowest detectable Br level was found in a PP/PE sample containing 57 mg kg^−1^ Br from TBBPA and decaBDE. For all Br-positive samples, with a Br level higher than 40 mg kg^−1^, TBBPA was detected in all seven samples as the most abundant BFR. In each Br-positive sample decaBDE and/or DBDPE were also detected. Only in one case was BTBPE detected in combination with TBBPA and DBDPE (ABS sample containing 279 mg kg^−1^ Br). Other target BFRs like TBBPA-DBPE, HBCD, PBBs and 2,4,6-tribromophenol were not detected in the samples. Interesting was the fact that no RoHS-regulated BFRs (European Commission 2002) were detected. The obtained data show that in some cases DBDPE as the newer replacement for decaBDE was found in the samples. Generally spiking the use of decaBDE and DBDPE, or other combinations of BFRs, together in one sample is quite rare and might confirm the presence of a mixture of different polymers. This suggestion was confirmed by identification of the main polymer matrix by ATR-FTIR combined with the detection of suspected macromolecular contaminants using ATR-FTIR and pyrolysis GC-MS. Surprisingly, in all cases macromolecular contaminants were detected. These detected polymer fractions were either based on styrene (ABS/PS/HIPS/SAN-type), acrylate (PMMA) or polyester (PET/PBT) but does not commonly appear in the main matrix unless as a recycled fraction. The detected macromolecular contaminants are fitting in with the literature claiming similar abundant polymer types in WEEE. No PA6 was found in the samples by the present methods.

**Figure 1.**
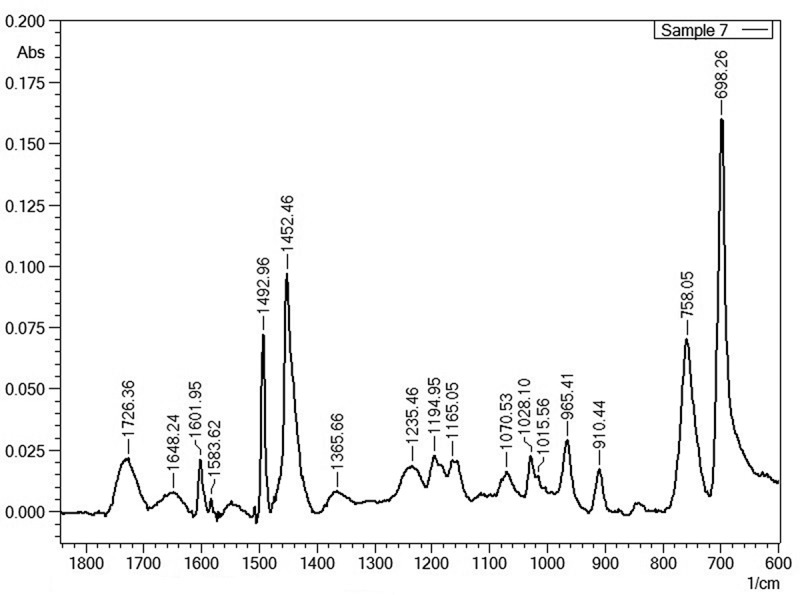
FTIR spectrum from sample 7 showing peaks from ABS (at 698.26, 758.05, 910.44, 965.41, 1028.10, 1452.46 and 1492.96 cm^−1^) as a majority with traces of polycarbonate (at 1015.56, 1070.53, 1165.05, 1194.95 and 1235.46 cm^−1^), traces of PP (at 1365.66 cm^−1^) and a weak ester peak at 1726.36 cm^−1^. This ester peak was confirmed by pyrolysis GC-MS belonging to methylmethacrylate from PMMA traces.

**Figure 2.**
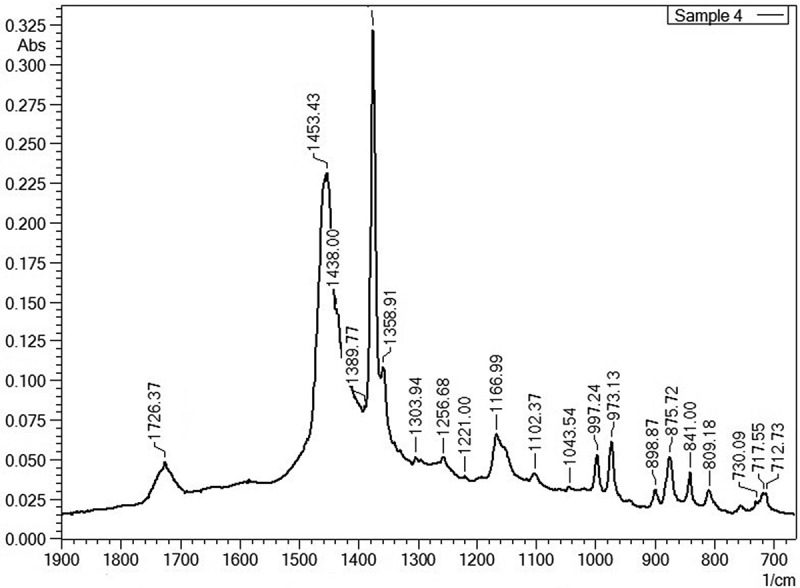
FTIR spectrum from sample 4 showing peaks from CaCO_3_ (at 712.73 and 875.72 cm^−1^) and PP/PE as the main matrix (at 717.55, 809.18, 841.00, 898.87, 973.13, 997.24, 1043.54, 1102.37, 1166.99, 1221.00, 1256.68, 1303.94, 1354.91, 1389.77, 1438.00 and 1453.43 cm^−1^) with an ester peak probably from PBT or PET (at 1726.37 cm^−1^). The peak at 730.09 cm^−1^ represents residual toluene from the sample extraction step for removal of BFRs.

**Figure 3.**
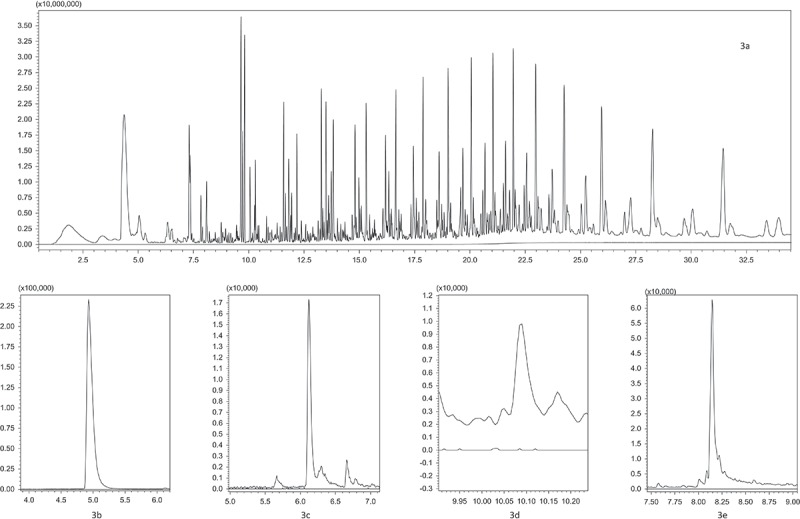
Full-scan pyrogram of sample 4 showing branched aliphatic hydrocarbon distributions from the PP/PE polymer matrix (a; full scan 50–1000 *m/z*); selected peaks of styrene (b; selected ions 78.0 and 104.1 *m/z*) and α-methylstyrene (c; selected ions 103.1 and 118.2 *m/z*) confirming the presence of polystyrene fractions in the sample; peaks of 1-phenyl-1-propene (d; selected ions 54.1/77.0 and 105.1 *m/z*) and benzoic acid (e; selected ions 77.0/105.1 and 122.1 *m/z*) prove the presence of PBT or PET traces in the sample. All pyrograms are compared with a sample blank which is represented by each chromatogram at the bottom.

Most information obtained from the ATR-FTIR data was confirmed by pyrolysis GC-MS measurements, however for the identification of macromolecular contaminants in some cases either ATR-FTIR or pyrolysis GC-MS were favoured for the detection of specific macromolecules or their pyrolysis products. ATR-FTIR has some advantages for the detection of traces of PC. PC was easier to detect by ATR-FTIR on typical wave numbers like 1015.6, 1070.5, 1165.1, 1195.0 and 1235.5 cm^−1^ as PC could not be measured by pyrolysis GC-MS due to the interferences of BPA potential coming from the debromination of TBBPA. The wave numbers selected by ATR-FTIR are specific for polycarbonate and do not suffer from interferences related to free BPA (Figure 1). Keeping this background in mind, PC was not a target analyte by pyrolysis GC-MS. On the other hand, styrenic co-polymers (HIPS, ABS, SAN), PBT/PET and PMMA were better detectable by pyrolysis GC-MS due to interferences in the ATR-FTIR data from the PP/PE matrix (see the FTIR spectrum in Figure 2 and the pyrogram in Figure 3) and mainly due to the presence of clear mass spectral data of monomers obtained after the unzipping reaction during pyrolysis which results in a higher certainty during the identification process. Beside the detection of BFRs and the macromolecular contaminants present in black FCAs, WEEE-relevant elements were also measured (Table 4). As a sampling strategy; the samples were acid digested in triplicate in order to avoid variations caused by potential inhomogeneity causing fluctuations in elemental content.

**Table 4.** Elemental composition of the samples.

Element	Sample 1 (mg kg^−1^)	Sample 2 (mg kg^−1^)	Sample 3 (mg kg^−1^)	Sample 4 (mg kg^−1^)	Sample 5 (mg kg^−1^)	Sample 6 (mg kg^−1^)	Sample 7 (mg kg^−1^)	Sample 8 (mg kg^−1^)	Sample 9 (mg kg^−1^)	Sample 10 (mg kg^−1^)
As	3.98 ± 0.21^a^	7.20 ± 0.24	0.54 ± 0.12	0.97 ± 0.03	4.05 ± 0.23	0.30 ± 0.04	7.39 ± 0.07	0.54 ± 0.01	0.57 ± 0.16	n.d.
Be	n.d.^b^	n.d.	n.d.	n.d.	n.d.	n.d.	n.d.	n.d.	n.d.	n.d.
Cd	2.03 ± 0.03	n.d.	5.85 ± 0.05	0.78 ± 0.04	5.50 ± 0.04	0.42 ± 0.01	11.93 ± 0.06	0.42 ± 0.04	1.08 ± 0.04	n.d.
Ce	8.94 ± 0.01	7.29 ± 0.01	n.d.	1.69 ± 0.01	n.d.	n.d.	n.d.	n.d.	n.d.	n.d.
Cr	19.41 ± 0.47	2.43 ± 0.14	1.38 ± 0.16	7.51 ± 0.56	6.54 ± 0.31	0.56 ± 0.18	8.55 ± 0.26	8.61 ± 0.04	14.01 ± 0.06	n.d.
Cu	37.30 ± 1.07	n.d.	10.99 ± 4.10	36.53 ± 0.72	20.13 ± 7.11	3.57 ± 0.80	75.97 ± 38.70	17.83 ± 0.85	48.23 ± 0.72	n.d.
Dy	0.42 ± 0.01	n.d.	n.d.	n.d.	n.d.	n.d.	n.d.	n.d.	0.26 ± 0.01	n.d.
Er	n.d.	n.d.	n.d.	n.d.	n.d.	n.d.	n.d.	n.d.	n.d.	n.d.
Fe	1207.12 ± 61.10	58.82 ± 3.98	65.92 ± 11.94	466.12 ± 17.04	75.29 ± 3.08	52.12 ± 5.17	124.79 ± 8.72	163.79 ± 10.58	466.46 ± 8.39	4.83 ± 2.66
Hg	0.14 ± 0.01	0.02 ± 0.01	0.36 ± 0.01	0.06 ± 0.01	0.81 ± 0.03	0.26 ± 0.01	0.76 ± 0.03	0.18 ± 0.01	0.02 ± 0.01	n.d.
La	2.40 ± 0.01	n.d.	n.d.	n.d.	n.d.	n.d.	n.d.	n.d.	n.d.	n.d.
Nd	2.51 ± 0.01	n.d.	n.d.	0.55 ± 0.13	n.d.	n.d.	n.d.	n.d.	0.28 ± 0.09	n.d.
Ni	2.99 ± 0.32	1.88 ± 0.08	7.29 ± 0.55	1.64 ± 0.37	2.35 ± 0.39	0.34 ± 0.25	6.93 ± 0.80	0.91 ± 0.10	1.41 ± 0.17	0.54 ± 0.21
Pb	99.36 ± 0.87	n.d.	7.75 ± 0.39	27.83 ± 0.93	26.49 ± 1.70	3.67 ± 0.68	38.79 ± 1.45	42.56 ± 0.82	63.86 ± 2.21	n.d.
Pr	4.54 ± 0.01	n.d.	n.d.	n.d.	n.d.	n.d.	n.d.	n.d.	n.d.	n.d.
Sb	n.d.	504.53 ± 73.50	52.50 ± 1.70	n.d.	113.53 ± 2.00	n.d.	270.87 ± 40.20	n.d.	n.d.	n.d.
Y	1.99 ± 0.01	n.d.	n.d.	0.56 ± 0.12	n.d.	n.d.	n.d.	n.d.	n.d.	n.d.
Zn	100.65 ± 2.08	29.78 ± 0.18	60.05 ± 1.11	70.48 ± 3.38	38.38 ± 0.27	23.25 ± 0.90	76.81 ± 1.23	47.98 ± 1.02	109.65 ± 1.16	24.51 ± 1.63

Notes: ^a^Uncertainty is expressed as the standard deviation *σ_n_*_–1_ of a triple measurement.

^b^n.d., Not detected with a value below the LOD for the selected element (an overview of the LOD values is given in Table 1).

For the evaluation, if WEEE was used for the production of black FCAs, Sb was presumed to be a key element. The results demonstrate that, in a majority of the cases, BFRs were present in combination with higher Sb concentrations. The concentration of Sb was in four of the seven cases higher in FCAs containing BFRs, which corresponds to the use of Sb_2_O_3_ as a synergist FR with halogenated FRs. In all cases, when Br was detected at elevated concentrations (> 200 mg kg^−1^ of Br), Sb was detected as well. As an example, sample 2, which is a PBT sample, did contain Br at a level of 5975 mg kg^−1^ joined with a concentration of 504 mg kg^−1^ of Sb. The presence of Sb in PBT or PET can be justified as Sb_2_O_3_ is commonly used as a catalyst in such matrices, however the presence of Sb and Br together is not common in PBT or PET applications unless to give flame retardancy. In this sample, it was expected that the addition of flame-retarded HIPS/ABS or SAN fractions is causing the undesirable presence of these contaminants.

In most of the BFR-positive samples typical elements used in electronic equipment like ferrous elements and the selected heavy elements (As, Cd, Cr, Cu, Fe, Hg, Ni, Pb and Zn) were present either at trace level or at elevated concentrations. The presence of these elements was expected as they appear in many applications within electrical and electronic equipment. Despite the expectations, for these elements no real difference can be seen between the ferrous and selected heavy element concentrations from samples positive for Br/BFRs and samples negative for Br/BFRs. The element Be was not detected at all in any of the measured samples, which reflects the actual situation that metal and composites containing Be are rarely used in consumer electrical and electronic equipment. Be-containing alloys are mainly used in consumer products like cellular phones (Grob et al. 2008). From these data it can be concluded that elemental contaminants might come from WEEE housings (Br, Sb) and small electronic PCBs but contamination from bigger metallic parts was not observed as Cd, Cr, Cu, Fe Ni, Pb and Zn concentrations stay below the 0.1 weight % level. Moreover the Cd, Hg Pb and total Cr concentrations in all samples were lower than the required RoHS limits for elements. Heavy elements like As and Hg were detected only at trace level (below 10 mg kg^−1^).

Typical REEs (Ce, Dy, La, Nd, Pr and Y) found in many electronic and electric applications nowadays were present in four of the seven Br-positive samples, while in Br-negative samples no traces of REEs were detected. Elements like Nd, which is a vital element in industrial batteries used in combination with Pr for the production of electric motors, were detected in three of the seven Br-positive samples. Combinations of Ce, Dy, La and Y mainly used in LED applications were detected in four of the seven Br-positive samples. Er, an active element in erbium-doped fibre amplifiers used in optical communication applications (Becker et al. 1999), was not detected in any samples.

## Conclusions

The purpose of this study was to obtain analytical data by combining several analytical techniques in order to prove the undesirable use of WEEE hidden in black polymeric FCAs sold on the European market. Therefore the selected target analytes were abundant organic and inorganic components which are appearing commonly in WEEE. At first the Br content was measured where for seven of the 10 selected samples Br was detected. Based on literature findings, the measured Br concentrations (between 57 and 5975 mg kg^−1^) appear too low to achieve flame retardancy sufficiently and therefore it is assumed that a small fraction of Br-containing polymers were used as a contaminant. The most abundant detected BFR was TBBPA, which was ever present in combination with either technical decaBDE or DBDPE. Additionally to these BFRs, in one sample BTBPE was also detected as the third BFR. The presence of BFRs in polymeric FCAs on the European market is prohibited (European Commission 2011a) and raw material contamination using recycled plastic fractions is suggested rather than the intentional use of flame-retarded polymers. Especially the fact that decaBDE and DBDPE were found in several samples together suggests the mixing of several polymer fractions. Within this study, the presence of foreign polymer fractions was checked where in all cases foreign macromolecular contaminants were detected. In some cases several macromolecular contaminants were detected in one sample.

Additionally, in order to confirm the root of contamination, WEEE-relevant elements were measured. Elements like As, Cd, Cr, Cu, Fe, Hg, Ni, Pb, Sb and Zn were present either at trace level or at elevated concentrations. Beside the presence of Br and BFRs, the presence of Sb is also a strong indicator for WEEE mixing. Concurrently Sb was detected with Br confirming the synergetic use of Sb in combination with BFRs; Sb was detected in all cases when Br was present at elevated concentrations (> 200 mg kg^−1^). This study described also the measurement of REEs and confirms in addition the suggested WEEE root of contamination. REEs like Ce, Dy, La, Nd, Pr and Y were detected in four of the seven BFR-positive samples.

To the best of our knowledge, this is the first paper reporting the presence of BFRs combined with the determination of REEs and other WEEE-relevant elements as WEEE precursors present in polymeric FCAs on the European market. The authors want to address their concern to the scientific community, policy-makers and control authorities by presenting real data about the abundance of WEEE-related chemicals in these products. Despite the fact that this study was carried out on a very small number of samples, it provides preliminary data to reflect the actual situation on the European market.
